# A highly reversible room-temperature lithium metal battery based on crosslinked hairy nanoparticles

**DOI:** 10.1038/ncomms10101

**Published:** 2015-12-04

**Authors:** Snehashis Choudhury, Rahul Mangal, Akanksha Agrawal, Lynden A. Archer

**Affiliations:** 1 School of Chemical and Biomolecular Engineering, Cornell University, 120 Olin Hall, Ithaca, New York 14853, USA

## Abstract

Rough electrodeposition, uncontrolled parasitic side-reactions with electrolytes and dendrite-induced short-circuits have hindered development of advanced energy storage technologies based on metallic lithium, sodium and aluminium electrodes. Solid polymer electrolytes and nanoparticle-polymer composites have shown promise as candidates to suppress lithium dendrite growth, but the challenge of simultaneously maintaining high mechanical strength and high ionic conductivity at room temperature has so far been unmet in these materials. Here we report a facile and scalable method of fabricating tough, freestanding membranes that combine the best attributes of solid polymers, nanocomposites and gel-polymer electrolytes. Hairy nanoparticles are employed as multifunctional nodes for polymer crosslinking, which produces mechanically robust membranes that are exceptionally effective in inhibiting dendrite growth in a lithium metal battery. The membranes are also reported to enable stable cycling of lithium batteries paired with conventional intercalating cathodes. Our findings appear to provide an important step towards room-temperature dendrite-free batteries.

## Abstract (web summary)

There is intensive research effort in suppressing lithium dendrite growth in lithium batteries. Here, the authors report the use of a crosslinked nanoparticle-polymer composite membrane with high mechanical strength and ionic conductivity which enables stable cycling of lithium metal batteries.

## Graphical abstract

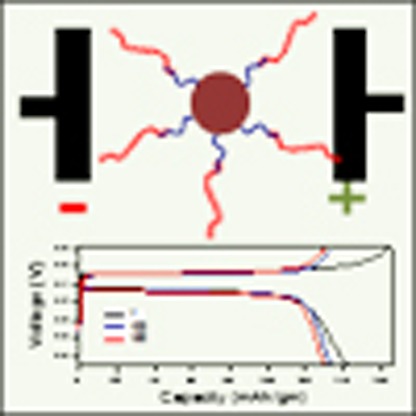

The search for portable, high capacity and safe electrical energy storage technologies remains one of the paramount motivators for materials research. High voltage cathodes, high energy anodes and highly conductive, but stable, electrolytes for lithium-ion batteries have received a lop-sided share of the attention by researchers because of their multiple attractive features, including high energy density, light weight, high operating voltage and minimal memory effects^1^,^2^,^3^. Secondary lithium metal batteries (LMBs), wherein metallic lithium serves as the anode, are an attractive alternative to lithium-ion batteries, but are known to have a serious problem associated with dendrite-induced short circuits^4^. During repeated cycles of charge and discharge, the uneven deposition of Li-ion on this metal lead to the formation of ramified structures, which grow unstably, punctures the separator and ultimately causes cell failure by short circuiting the anode and cathode. Over the years, a growing number of studies have explored electrolyte and separator platforms to suppress the dendrite growth in an effort to enable LMBs^5^,^6^. Recent efforts have focused on stabilizing the surface of the Li anode using electrolyte additives^7^,^8^,^9^, hybrid ionic liquid nanostructures^10^,^11^ or by using a high modulus separator^12^,^13^,^14^, which can also provide a means of applying compression forces to stabilize the anode during deposition. Infusion of a nanoporous ceramic or polymer membrane separator with a liquid electrolyte that can facilitate Li ion transfer, without compromising the mechanical properties of the nanoporous membrane, provides a more straightforward route towards mechanically strong, room-temperature electrolytes/separators that prevent dendrite growth^15^,^16^.

Solid or gel-polymer electrolyte have been researched extensively for their ability to enable batteries in various form factors that are leakage free, flexible, yet safer^12^,^13^,^14^,^17^,^18^,^19^,^20^,^21^. However, these gel electrolyte system have consistently underperformed in terms of the ionic conductivity requirements for room-temperature operation of advanced batteries^22^. In a block copolymer solid electrolyte, the ratio of the hard non-conducting phase to the soft conducting phase determines the mechanical strength. It has been shown for instance that in poly(styrene)-poly(ethylene oxide) (PS-PEO) electrolytes, a PS/PEO molar ratio of around unity provides a good balance between mechanical strength and ionic conductivity^23^. However, the abundance of the non-conducting, reinforcing PS phase still results in low bulk conductivity relative to liquid electrolytes, necessitating elevated temperature battery operation, which is a limitation for many consumer-based applications. Nanocomposite electrolytes comprised of liquid or polymeric electrolytes reinforced with nanoparticle fillers can achieve higher modulus at lower reinforcing material content, which potentially offers multiple straightforward paths towards electrolytes with high modulus and acceptable room-temperature ionic conductivity^24^,^25^,^26^,^27^,^28^. Uniform dispersion of fillers in polymer host is understood to be a prerequisite to prevent particle agglomeration and local inhomogeneity in the electrolyte medium. Unfortunately, strong attractive van der Waals and depletion forces exerted on the particles by their polymer host result in particle aggregation and phase separation^29^,^30^. Several recent studies have shown that various physical and chemical modifications of nanoparticle-polymer interactions can lead to dramatic improvements in phase stability and electrolyte properties of such systems^29^,^31^,^32^,^33^,^34^.

A strategy towards a hybrid electrolyte platform, which can provide high ionic conductivity and attractive mechanical properties, is to design a crosslinked polymer web in which hairy nanoparticles serve as crosslinkers. A perhaps obvious benefit of this design is that chemistry introduced on the surface of the precursor particles can be presented in the pores of the crosslinked material to selectively pass Li ions while hindering the unstable dendrite growth. Here we report the realization of this concept and show that an electrolyte with the proposed design combines the advantages of a nanocomposite and solid polymer electrolyte. In the systems reported herein, a hydrophobic polymer provides a porous conductive pathway for Li-ion migration in a liquid electrolyte phase, while a short hydrophilic oligomer tethered to the nanoparticle surface constrain the network providing structure and mechanical strength. The nanoparticle-induced crosslinking of the polymer prevents particle aggregation, which is known to compromise flexibility and elasticity of nanocomposites.

## Results

### Synthesis and physical characterization of crosslinked membranes

**Figure 1:**
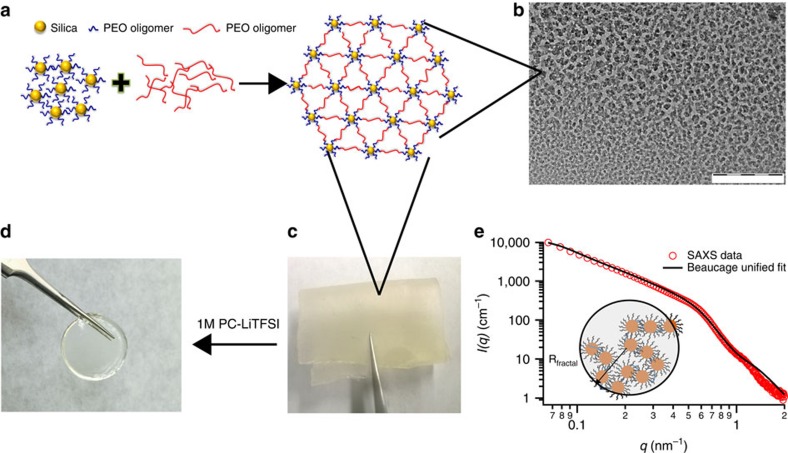
From hybrid nanostructure to macroscale membrane: (**a**) The scheme of reaction involved in the synthesis of the free-standing crosslinked nanoparticle-polymer composite. Silica tethered with hydroxy-terminated polyethylene oxide is reacted with polypropylene oxide-diisocyanate with a weight ratio of 1:4 at room temperature in a teflon mould of appropriate shape. (**b**) TEM image of the crosslinked membrane. The black circles are silica particle dispersed in the polymer matrix. Scale bar, 200 nm. (**c**) Photograph of a free-standing membrane. Physically it has a rubbery texture. (**d**) Image of the wet polymer gel obtained by long-time soaking of the prepared membrane in unimolar electrolyte liquid. (**e**) Scattered intensity *I*(*q)* versus wave vector *q* profile obtained from SAXS measurements. Solid line represents the fit for Beaucage Unified model to the data. The fit in the low *q* regime shows a power-law scattering (*I*(*q*)∼*q*^−*α*^) with an exponent (*α*) of ∼2, indicating the presence of mass fractals.

Figure 1a is a schematic of the reaction steps involved in the synthesis of our crosslinked hybrid electrolyte material. First, short-chained oligomers, PEO—500 Da, are grafted onto silica particles to form nanoparticle organic hybrids with reactive end groups. The PEO-tethered silica particles are next linked with difunctional poly(propylene oxide)—2,000 Da (PPO) to form the crosslinked nanoparticle-polymer composite (CNPC) in a desired macroscopic shape. The materials used in the present study achieve high levels of crosslinking by two-reaction mechanisms: dimerization between isocyanate end groups in PPO, and reaction between isocyanate group in PPO and end-functional hydroxyl groups on the hairy nanoparticles to form stable urethane linkages. The CNPC membrane (Fig. 1c) is mechanically tough and has a ‘rubbery' texture. To use it as a composite electrolyte, the membrane was soaked in a liquid electrolyte comprised of propylene carbonate (PC) containing 1 M lithium bis(trifluoromethanesulfonyl)imide (LiTFSI) salt for a period of 2 days. This step yields a material with a swollen, gel-like appearance (shown in Fig. 1d), but with little loss of the solid nanocomposite material's mechanical strength. In contrast, it is seen that on soaking with electrolyte, the un-crosslinked components separate out in the liquid, leaving behind a transparent film with no observable particle aggregates. For brevity, the CNPC membranes infiltrated with liquid electrolytes are hereafter referred to as CNPC electrolytes.

The reaction progress can be facilely mapped using Fourier transform infrared spectroscopy (FTIR) as illustrated in Supplementary Fig. 1. It is evident that the -NCO bond at (2,270 cm^−1^) present in the neat PPO-diisocyanate polymer is consumed in the reaction and at same time generates a urethane linkage, evident by the -NH vibration (3,288 cm^−1^) and -C=O bond peak (1,680 cm^−1^). TGA analysis of these materials (Supplementary Fig. 2) shows that the freestanding crosslinked film contains 6% silica by weight and in the final CNPC electrolyte material the silica content is 2% (Supplementary Table 1), thus there is a preponderance of ion-conducting entity in the material. Significantly, because the silica particles are nano sized (∼10 nm) and have multiple chains attached to their surface (∼55 chains per particle), each particle provides a large number of node points for crosslinking the PPO polymer. Thus, even a low particle content in the precursor material is expected to enable extensive crosslinking via particle nodes, which should lead to dramatic improvements in mechanical properties without compromising room-temperature ionic conductivity of the liquid electrolyte hosted by a CNPC membrane.

The synthesized CNPC membranes were characterized using differential scanning calorimetry measurements (Supplementary Fig. 3), which reveal a low glass transition temperature (*T*_g_) of −63 °C for neat PPO polymer; after crosslinking *T*_g_ increases to −42 °C. The dispersion and arrangement of nanoparticles in the crosslinked polymer matrix is important for the targeted application as nanostructured electrolytes. Figure 1b shows a 2D image of a thin layer of the material obtained from transmission electron microscopy (TEM). The image shows that the particles organize in disordered inter-connected, string-like phases to produce tortuous pores with an average diameter of 24±2 nm estimated from the TEM micrograph (see Supplementary Fig. 4). Small angle X-ray scattering (SAXS) has been used previously to study particle packing and polymer-nanoparticle interactions in bulk nanocomposite-based electrolyte materials^35^,^36^,^37^. Figure 1e shows the experimental intensity *I*(*q*) as a function of wave number (*q*) obtained from SAXS measurements on CNPCs. The solid line through the experimental *I*(*q*) data was obtained using the Beaucage Unified model (see Supplementary Methods). The power-law scattering regime *I*(*q*)∼*q*^−*α*^, with exponent *α*≈2 observed at low *q* indicates that the particles in the materials are organized in the form of mass fractals with sizes in the range 50–70 nm. The shoulder at *q*=*q*_*s*_≈0.6 nm^−1^ implies that the scattering originates from particles with average diameter *d*=2*π*/*q*_*s*_≈10.5 nm, which is close to the average diameter of SiO_2_ nanocores used for synthesizing the CNPC. Finally, the absence of any additional structure contribution (maxima) in *I*(*q*) in the intermediate and high *q* regime means that the primary particles are reasonably far apart. The interparticle distance can be theoretically calculated assuming random packing of hard spheres in suspension, *d*_p–p_=*d*(0.63/*φ*)^1/3^, where *d* is the nanoparticle diameter and *φ* is the volume fraction of particles in the polymer network. This analysis yields an interparticle spacing of ∼27 nm, which is comparable to the particle separation of 24±2 nm deduced from analysis of the TEM image in Fig. 1b (see Supplementary Fig. 4). Thus, the TEM and SAXS results imply that in a freestanding CNPC membrane, randomly distributed particles organize in string-like phases to form tortuous path for ion and mass transport through the membrane. Such nanostructured materials have been hypothesized, largely without proof, to be excellent candidates to hinder dendrite-induced short circuit in lithium batteries^13^,^14^,^20^.

### Mechanical and electrochemical properties

**Figure 2:**
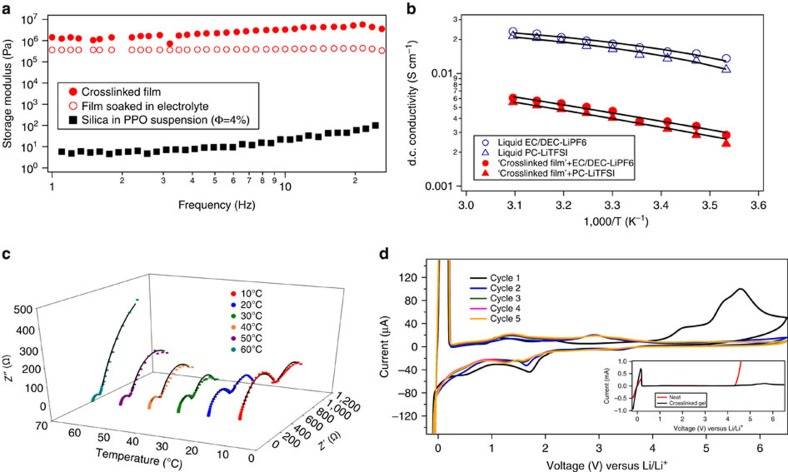
Good mechanical and electrochemical properties: (**a**) Storage modulus (Pa) as a function of frequency, comparing the dry polymer network and gel electrolyte with a suspension of silica in PPO polymer with same volume fraction. The modulus of crosslinked gel is 5 orders of magnitude higher than the suspension. (**b**) DC Conductivity as a function of inverse absolute temperature. The markers show measured values, while the continuous lines are fitted Vogel Tamman Fulcher curves. The crosslinked gel shows conductivity an order of magnitude less than the intrinsic value of eletrolyte. (**c**) Impendence spectroscopy results for a symmetric lithium battery, with crosslinked gel electrolyte plotted against temperature, where the *x*-axis denote temperature, *y*-axis denote real impedance (ohms) and *z*-axis denote imaginary impedance (ohms). (**d**) Cyclic voltametry of a cell with configuration Li/crosslinked gel/stainless steel for 5 cycles. The inset compares the stability of the crosslinked gel with neat electrolyte of 1 M PC-LiTFSI.

Figure 2a reports the storage modulus as a function of frequency for a freestanding CNPC film before and after soaking in liquid electrolyte. The storage modulus of a suspension of PPO in silica is also shown in the figure. Clearly, crosslinking increments the modulus by several orders of magnitude. The mechanical strength of the material is about 5 orders of magnitude higher than a neat PEO-based electrolyte, and is comparable to the elastic shear modulus reported for dry PS-PEO block copolymer-based electrolytes^23^,^38^. In addition, it is seen that even after soaking in a liquid electrolyte, the elastic modulus of the CNPC material remains high; it is higher than those recently reported for crosslinked PEO–PE–PEO solid polymer electrolytes^14^. This latter feature we attribute to the large number of crosslinks made to a single particle and the short length and stiffness of the polymer fragments used as crosslinkers. Figure 2b reports the direct current conductivity in Arrhenius plot for CNPC electrolytes obtained by soaking the as synthesized CNPC membranes in two widely studied liquid electrolytes, PC-LiTFSI and EC/DEC-LiPF_6_. In comparison to the neat (no nanoparticles) liquid electrolytes, the conductivity is lower, by about 1 order of magnitude, but is still high enough at room temperature for most lithium battery applications. This high conductivity is attributed to the low glass transition temperature of PPO and the low fraction of non-conductive nanoparticles needed to produce materials with high mechanical strength. The conductivity versus temperature data is well-described by the Vogel Tamman Fulcher relationship *σ*=*Α* exp(−*B*/(*T*−*T*_O_)), where *A* is the pre-exponential factor corresponding to conductivity at infinite temperature, *B* is the activation energy and *T*_O_ is the reference temperature. The respective coefficients for the different systems are tabulated in Supplementary Table 2.

Figure 2c reports temperature-dependent impedance spectra for CNPC electrolytes obtained by soaking the membranes in PC-1MLiTFSI electrolyte. The measurements were performed in symmetric (Li|Li), two-electrode cells. Solid lines through the data were obtained using an equivalent circuit model previously reported for nanocomposite-based electrolyte systems^39^,^40^,^41^. The bulk and interfacial resistance deduced from the Nyquist plots are plotted as a function of temperature in Supplementary Fig. 5. It is evident that both resistances decrease with temperature and the bulk resistance is always lower than the interfacial resistance. This implies that ion transport through the tortuous materials is in reality easier relative to ion transfer across the electrolyte/electrode interface. It is apparent nonetheless that the bulk and interfacial resistances (Supplementary Fig. 5) exhibit similar dependence on temperature, indicating that both have similar activation energy. It can therefore be concluded that the CNPC electrolytes make good contact with the Li metal, as just the polymer itself appears to limit ion transport across the interface. The electrochemical stability for the CNPC electrolyte was analysed at room temperature using cyclic voltammetry in a prototype cell with the following configuration, Li|CNPC+PC-1MLiTFSI|stainless-steel. Measurements were performed between −0.2 to 6.5 V and a scan rate of 1 mV s^−1^ was employed. Figure 2d shows the current as a function of Voltage for the CNPC electrolyte at different cycle numbers, while the inset shows the equivalent first cycle results obtained using a PC-1MLiTFSI liquid electrolyte. The significant current peaks at ∼−0.2 V versus Li^+^/Li and +0.2 V versus Li^+^/Li indicate the plating and stripping of Li ions onto/from stainless steel^42^. As previously reported^43^, a passivation layer is formed on the Li metal surface at 4.1 V versus Li^+^/Li, indicated by the current peak, which disappears in subsequent cycles. The CNPC electrolyte also shows a high stability window, with cathodic stability of at least 5 V versus Li^+^/Li, which is superior to what is observed for a PC-LiTFSI liquid electrolyte.

### Stability analysis of lithium electrodeposition

**Figure 3:**
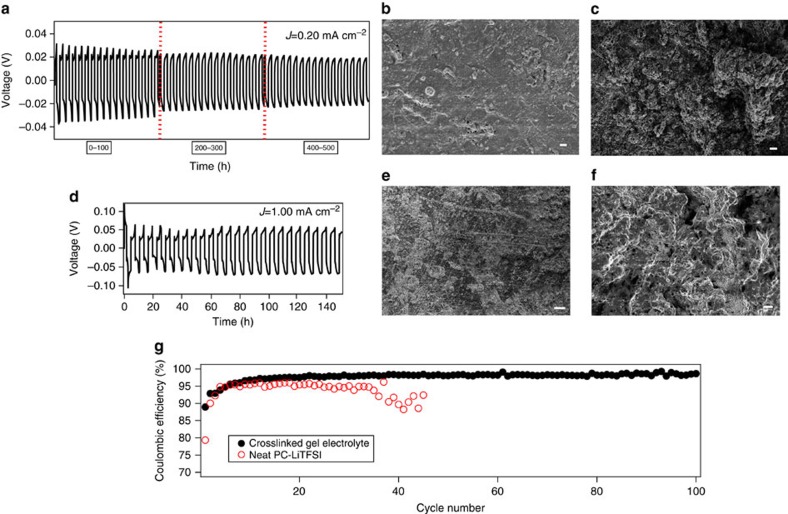
Inhibiting dendrites and enabling smooth electrodeposition: (**a**) Plate-strip cycles for a symmetric cell comprising of crosslinked gel electrolyte is shown at a current density of 0.20 mA cm^−2^, showing stable performance for over 500 h. (**b**) SEM image of lithium metal anode after 100 h of cycling with the crosslinked gel electrolyte is shown, where very smooth surface is observed. (**c**) Morphology of lithium metal anode with neat electrolyte is shown, where dendritic structures of lithium is seen to have formed. (**d**) Volatge versus time plots at 1.00 mA cm^−2^ for crosslinked gel-based cells are shown, the cells show no sign of short circuit for at least 120 h. (**e**) The post mortem analysis for crosslinked gel electrolyte. (**f**) Lithium surface with neat electrolyte after 100 h of cycling shows dense dendrites that are capable of shorting the cells. All white scale bars measure 20 μm. (**g**) Electrochemical tests with Li| electrolyte| stainless-steel configuration at a current density of 0.25 mA cm^−2^ comparing coulombic efficiency as a function of cycle numbers for pristine and crosslinked gel electrolytes with 2%(vol.) V.C. and 1%(wt.) LiNO_3_ additive.

Figure 3a,d report results from a so-called ‘strip-plate test' in which symmetric Li|Li cells are charged and discharged sequentially for a period of three hours. The voltage profiles for liquid electrolytes are shown in Supplementary Fig. 6. It is clear that cells containing the CNPC electrolyte exhibit remarkably high cycling stability in comparison to standard cells with the same liquid electrolyte infused in the CNPC membrane. At a current density of 0.20 mA cm^−2^, the cells show stable voltage profiles for more than 500 h. In comparison, a control symmetric cell with the same electrolyte and without the crosslinked membranes fails within 60 h (see Supplementary Fig. 6). At a higher current density of 1 mA cm^−2^, the cells run for more than 120 h, whereas, the voltage profile for the control cell is already unstable from the first cycle of the test. Figure 3 reports the morphology of the lithium anode surface using Scanning Electron Microscopy (SEM) after 100 h of cycling for both types of cells discussed above. Supplementary Fig. 7 shows the image of a pristine uncycled lithium electrode. At a current density of 0.20 mA cm^−2^, the anode surface is smooth with only sporadic patches of rough deposits (Fig. 3b) for cells based on the CNPC electrolyte. In contrast, clear sharp dendrites are formed on the surface of the anode cycled in control liquid electrolyte (Fig. 3c). For cells cycled at higher current density of 1 mA cm^−2^ the SEM images show that after 100 h, a dendritic structure is visibly present on the anode surface cycled in both the CNPC and control liquid electrolytes, however, the control cells display comparatively sharper and much larger dendrites.

In a LMB, electrolytes are also prone to degradation as a result of parasitic chemical and electrochemical reactions with the reactive lithium metal anode. Uneven electrodeposition exacerbates this failure mode by creating fresh surface for additional reactions. The coulombic efficiency is an important parameter that allows these effects to be quantified and tracked from cycle to cycle; it can therefore be considered a surrogate measure of the stability of Solid Electrolyte Interface (SEI) on the lithium metal anode. Several recent studies have considered methods for improving the stability of the SEI layer using protective films on Li anodes^44^,^45^. Procedures ranging from deployment of sacrificial, surface-reactive additives in liquid electrolytes^7^,^9^,^46^,^47^,^48^, to use of electrolytes saturated with salt^49^ have been reported to be effective in stabilizing lithium metal.

We performed cycling studies in which a fixed amount of Li is transferred from a lithium metal electrode onto a stainless-steel substrate and quantify the recovery in the reverse process when Li is stripped from the substrate to determine the coulombic efficiency. Understanding that surface protection of lithium is required for meaningful coulombic efficiencies in LMBs, the control measurements were performed using liquid electrolytes comprised of PC containing 1 wt% LiNO_3_, and 2 wt% vinylene carbonate (VC) as an additive, which are reported to form a stable SEI on Li^48^. In contrast to the symmetric cell studies discussed in the previous section where reasonable cycling was possible in liquid electrolytes in cells using O-ring style separators, we discovered that the poor adhesion between deposited lithium and the stainless-steel counterelectrode caused lithium to separate and become electrically disconnected from the stainless-steel substrate, resulting in poor columbic efficiencies. Replacing the O-rings with a conventional Celgard separator eliminated this problem.

The coulombic efficiency obtained in this manner is plotted in Fig. 3g as a function of cycle number at a current density of 0.25 mA cm^−2^ for the control liquid electrolyte and for CNPC electrolytes (CNPC membranes soaked in the control liquid electrolyte). A typical voltage-capacity curve measured at the 40th cycle of these measurements is provided in the Supplementary Fig. 8. PC is known to form a very unstable SEI layer, yielding a low coulombic efficiency (∼75%)^50^, however, it is evident that the additives improve the efficiency to over 90% both in liquid PC and in the CNPC electrolytes. Nevertheless, it is seen from Fig. 3g that the neat electrolyte fails to sustain high coulombic efficiency beyond 45 cycles, while the CNPC electrolytes maintain high coulombic efficiency for at least 100 cycles. This can be rationalized by our earlier observation of smoother electrodeposition in the latter electrolytes, which helps in preventing the rupture and reformation of SEI layer in successive charge and discharge cycles.

**Figure 4:**
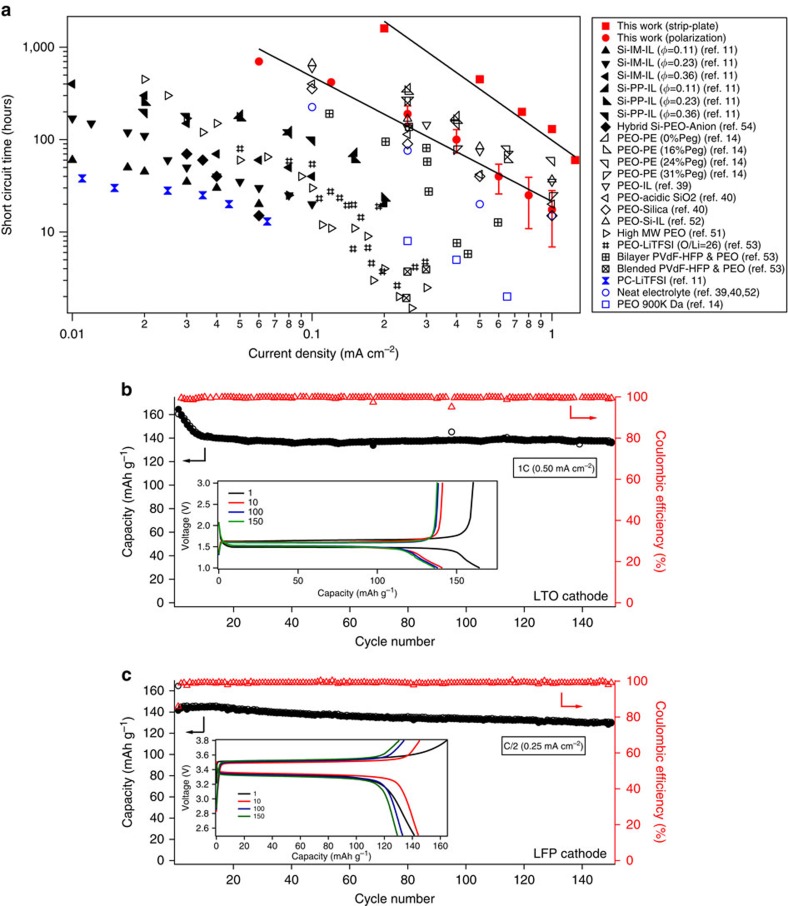
High short circuit time and good cyclic stability: (**a**) Short circuit time of crosslinked gel electrolytes compared with other state of art battery performance. Red squares and red circles indicate the Tsc for strip-plate test and polarization test in this work, respectively. The black filled symbols represent polarization tests done at room temperature, while the open symbols represent elevated temperature experiments. Black closed triangles represent silica tethered with imidazolium (Si-IM-IL) and piperidinium ionic liquid (Si-PP-IL) at various volume fractions of silica, indicated in parenthesis^11^. Black closed diamonds indicate anion tethered hybrid silica nanocomposites^54^. The high temperature data include crosslinked PE-PEO solid polymer with different plasticizer content given in parenthesis^14^. Other data points are PVdF-HFP/PEO composite^53^, high molecular weight polymer^51^, silica –polymer composite^40^, polymer with ionic liquid^39^ as well as their combination^52^. The blue symbols indicate neat/pristine electrolyte systems. Error bars denote deviations from different measurements. (**b**) Cycling performance for Li| crosslinked gel| LTO at 1C (0.50 mA cm^−2^). The inset shows the voltage profiles of the same. (**c**) Cyclic performance for Li| crosslinked gel| LFP at C-rate of C/2 (0.25 mA cm^−2^), with inset showing voltage profiles. In Figure **b** and **c**, closed black symbols indicate discharge capacity, open black indicate charge capacity. The red triangles denote coulombic efficiency.

Polarization experiments at a fixed current density provide a more aggressive protocol for evaluating stability of lithium electrodeposition. Figure 4a compares the cell lifetimes obtained using strip-plate and polarization measurements. A typical voltage versus time curve is provided as Supplementary Fig. 9. It is again apparent that the CNPC electrolytes enable cells that deliver over two orders of magnitude improvement in lifetime. The short circuit times (*T*_SC_ ) deduced for both the polarization test and strip-plate cycling studies can be fitted with a power-law function of the form, *T*_SC_=A.*J*^−K^, where *J* is the current density and A is a transport coefficient. The power-law exponents for the strip-plate and polarization measurements are, respectively, 1.84 and 1.35; consistent with previous reports^14^,^43^.

Figure 4a compares the short circuit time from our plate-strip and polarization experiments with results from a wide variety of literature studies. For example, Rosso *et al*.^51^ studied the stability of lithium electrodeposition in high-molecular-weight PEO containing lithium salt at 90 °C. The figure shows that at equivalent current densities, the crosslinked nanocomposite electrolyte based cells register substantially higher short circuit times in comparison to those obtained in the polymer electrolytes studied by Rosso *et al*. Figure 4a also reports results from the work of Liu *et al*.^39^,^40^,^52^ who performed electrochemical experiments in visualization cells in which surface modified silica-PEO-based nanocomposite electrolytes, solid polymer electrolytes comprised of PEO × LiTFSI repeating units, and combinations of the two were used to stabilize deposition in LMBs at elevated temperature; from Sannier *et al*.^53^ who performed symmetric cell studies using gel-polymer membranes based on PEO and PVdF-HFP polymers; and from Khurana *et al*.^14^ using crosslinked PEO–PE–PEO polymers. It is important to note that all of these experiments were performed at elevated operating temperature between 60 and 90 °C to overcome the poor room-temperature ionic conductivity of the electrolyte materials. It is apparent from the figure that the CNPC electrolytes deliver as good or better performance than all of these other studies with the important difference that the experiments the CNPC electrolytes were all performed at room temperature. Finally, Fig. 4a reports results from simple liquid electrolytes (here termed Neat electrolytes) and other state-of-art nanocomposite and polymer based electrolytes reported previously to be effective inhibitors of dendrite growth in LMBs operated at room temperature. Comparison of these results with those obtained in the present study also show that cells that utilize CNPC electrolytes exhibit superior ability to stabilize electrodeposition of Li than any of the other room-temperature electrolyte/separator materials^11^,^54^. On the basis of these results, we therefore conclude that the CNPC electrolytes reported in the present study are promising candidates towards the goal of dendrite-free room-temperature LMBs.

To further characterize the galvanostatic performance of our electrolyte materials, LMBs were constructed in which metallic lithium was paired with lithium titanium oxide (LTO) and lithium iron phosphate (LFP) as the cathode. For these studies, CNPC membranes were soaked in the commonly used EC/DEC (1v:1v) electrolyte solvent mixture containing 1 M LiPF_6_ salt. Again the CNPC electrolytes exhibited mS cm^−1^ level room-temperature ionic conductivities, allowing the batteries to be operated at room temperature. For practical applications, it is important for a battery to have high capacity for large number of cycles, even at high current densities. Figure 4b reports the cycling performance of Li|CNPC+EC:DEC(1MLiPF_6_)|LTO cells at a current density of 0.5 mA cm^−2^. The batteries retain high capacity for at least 150 cycles. Similar effect is observed at a high current density of 1.0mAcm^−2^ as shown in Supplementary Fig. 10. Figure 4c reports the performance of a battery where we paired metallic lithium with a LFP cathode in the CNPC electrolyte. At a current density of 0.25 mA cm^−2^, the battery retains a capacity of over 120 mAh cm^−2^ up to 150 cycles. Similar stable performance of the LFP based battery at 0.17mA cm^−2^ can be seen in Supplementary Fig. 11 inset of figures shows the voltage profiles of the respective cells exhibit clear plateaus and low IR losses. These results clearly show that the CNPC electrolytes have good compatibility with lithium metal and with conventional high-performance intercalating cathodes and anodes. This makes them promising candidate materials for LMBs in which a metallic lithium anode is paired with conventional intercalating cathodes.

## Discussion

Our findings provide a novel pathway towards nanostructured membranes in which chemistry introduced on the surface of nanoparticles can be internalized in the pores for regulating ion and mass transport. Here we illustrate the approach using hairy nanoparticles that can be crosslinked with rigid polymers to create ion-conducting membranes with good mechanical properties. Because each particle is functionalized with up to 55 reactive groups, this allows one to achieve highly crosslinked materials at very low particle contents. This makes it possible to create electrolyte membranes with both high mechanical modulus (*G*_*N*_=1 MPa) and high, liquid-like ionic conductivity (*σ*_o_=5 mS cm^−1^) at ambient temperature, where traditional high modulus electrolyte systems fail to maintain high conductivity. The crosslinked polymer-nanoparticle composite (CNPC) electrolytes are shown to be exceptionally effective in promoting smooth, dendrite-free electrodeposition of lithium metal at intermediate current densities. Comparison of the lithium metal anode lifetimes achievable in the new materials with those reported for other polymer, block-copolymer, crosslinked polymers and polymer-nanoparticle composites show that CNPC systems are the most promising for room-temperature LMB systems. Further, it is shown that these materials work efficiently in LMBs based on low-voltage LTO and intermediate voltage LFP cathodes, where they can be reversibly cycled with high discharge capacity for over 150 cycles, at high current densities.

## Methods

### Materials

Ludox SM30 colloidal silica (*d*=10±2 nm), poly(propylene glycol)-toluene-2,4-diisocyante, PC, bis(trifluoromethane) sulfonamide lithium salt, ethylene carbonate/ diethylene carbonate (1v:1v) with lithium hexafluorophosphate were all purchased from Sigma Aldrich. Hydoxy terminated poly (ethylene oxide)-silane was obtained from Gelest. All the chemicals were used as received in appropriate conditions.

### Nanoparticle-polymer crosslink synthesis and composite electrolyte preparation

Crosslinked-Nanoparticle-Polymer-Composites (CNPC) were synthesized using a two-step process. In the first step, colloidal silica was grafted with hydroxy-PEO-silane (500 Da) in water by the reaction of silanol groups of the PEO chain and OH- groups on the silica particle. A predetermined amount of the Ludox SM30 and a large excess of the hydroxy-PEO-Silane was first heated at 80 °C for a period of two days, before centrifuging the thus obtained mixture in a chloroform-hexane mixed solvent five times at a speed of 8,500 r.p.m. for 10 min to remove all unlinked PEO from the hairy nanoparticles. The recovered OH-PEO-SiO_2_ hairy particle suspension was rigorously dried and saved for the next reaction step. In the second step, poly(propylene glycol)-toluene-2,4-diisocyante and the OH-PEO-SiO_2_ nanoparticles were combined in a 4:1 ratio by weight and dissolved in anhydrous chloroform using a vortex mixer. The obtained solution was casted onto a rectangular Teflon mould of desired size. The mixture was allowed to crosslink overnight under ambient conditions to produce CNPC films/membranes of desired thicknesses. The resultant membranes were characterized using TGA, differential scanning calorimetry and FTIR. To use the synthesized CNPC membranes as battery electrolytes, they were soaked for a period of two days in conventional liquid electrolytes in an argon-filled glove box.

### TEM and SAXS

The nanoscale structure of the as prepared CNPC membranes was characterized using a FEI T12 Spirit TEM. For these measurements, a thin layer of the precursor material was cast onto the TEM grid, allowing it to crosslink on the grid. Before TEM imaging, coated grids were dried overnight using the same procedure used to prepare the freestanding CNPC membranes. SAXS measurements were performed at the Cornell High Energy Synchotron Source (CHESS) on a strip of the CNPC film using a point collimated X-ray source at room temperature.

### Mechanical properties

Mechanical properties of CNPC membranes and CNPC electrolytes were analysed using dynamic mechanical analysis (DMA Q800) at room temperature. A fixed strain of 0.1% was applied on the strip over a range of frequency of 1 to 100 Hz, to obtain the storage modulus of the samples. A similar experiment was done on the Silica-PPO suspension using an Anton Paar MCR501 shear Rheometer to characterize the effect of crosslinking on mechanical properties of the materials.

### Electrochemical characterization

The ionic conductivity of CNPC electrolytes was measured as a function of temperature using a Novocontrol N40 Broadband Dielectric instrument. The d.c. conductivity was obtained from the plateau, high frequency region of the a.c. conductivity versus frequency data. Impedance Spectroscopy measurements were performed using a Solatron frequency analyzer. A two-electrode, symmetric Li|Li cell in which the CNPC electrolyte was sandwiched between lithium foils was studied in a frequency range from 10^−3^ to 10^7^ Hz. Linear sweep and cyclic voltammetry were performed at a sweep rate of 1 mV s^−1^ between −0.2 to 6.5 V using Li/CNPC electrolyte/stainless-steel cells to quantify the voltage stability window of the electrolytes.

### Cell lifetime and failure studies

Symmetric 2032 type Li|Li coin cells containing either liquid of CNPC electrolytes were prepared inside an argon-filled glove box. To facilitate comparisons, all cells used in this component of the study also included a standard Celgard separator, with a diameter of 6.4 mm. The cells were evaluated using both galvanostatic (strip-plate) cycling and in step current (polarization) modes over a range of current density using a Neware CT-3008 battery tester. In the ‘strip-plate' experiments, the cells were charged and discharged at a predetermined current density for a period of 3 h each. Failure was deduced either from a sudden drop of the resultant voltage waveform or when the waveform exhibited new harmonics and displayed irregular time dependence. In the polarization experiments, the symmetric cells were constantly charged at a particular current density until the cell failed. In this case, failure was determined either by a sudden drop in the resultant voltage profile or by the appearance of irregular transients in the voltage profile. Postmortem characterization of the lithium surface after cycling was used to corroborate our conclusions about failure from the electrical response of the cells and for assessing the surface structure of Li in cells that showed no electrical signatures of failure. For this purpose, cells were dissembled in the glove box and the Li foil removed and was washed repeatedly in pure PC to remove the electrolyte, before performing SEM (LEO155FESEM) analysis.

### Measuring the coulombic efficiency

Li| electrolyte|stainless-steel 2032 type coin cells were assembled in an argon-filled glove box. Control experiments using liquid electrolytes comprised of 1 M LiTFSI-PC with 1%(wt) LiNO_3_ and 2%(vol.) vinylene carbonate additives and a standard Celgard separator were compared with measurements using CNPC electrolytes created by soaking CNPC membranes in these same liquid electrolytes. In both cases, prior to the measurements, cells were conditioned by cycling them between 0 and 0.5 V for 10 cycles, following a procedure reported in literature^44^ thought to enable formation of a stable SEI layer on the electrodes. To characterize the columbic efficiency, the conditioned cells were first discharged at a constant current density of 0.25 mA cm^−2^ for 2 h to transfer an amount of lithium corresponding to 0.50 mAh cm^−2^ of charge from the Li electrode to the stainless-steel electrode. The amount of charge recovered in the reverse cycle when the cell is charged back to 0.5 V at the same current density was recorded, and the fractional recovery used to determine the Columbic efficiency.

### Half-cell testing

Both LTO and LFP cathodes were prepared by mixing the active materials with Super P carbon and PVDF binder in the ratio of 8:1:1, by weight, in the presence of NMP, and casting a layer of the resultant slurry onto aluminium foil. Next, the cathode was dried first at room temperature and then at 70 °C overnight under vacuum. The surface capacity of both the films was maintained constant as 0.5 mAh cm^−2^. Li|CNPC electrolyte|LTO- and Li|CNPC electrolyte|LFP 2032-type coin cells were assembled under argon environment in a glove box.

## Additional information

**How to cite this article:** Choudhury, S. *et al*. A highly reversible room-temperature lithium metal battery based on crosslinked hairy nanoparticles. *Nat. Commun.* 6:10101 doi: 10.1038/ncomms10101 (2015).

## Supplementary Material

Supplementary Information Supplementary Figures 1-11, Supplementary Tables 1-2, Supplementary Methods and Supplementary Reference
