# Supplementary material for: A highly reversible room-temperature lithium metal battery based on crosslinked hairy nanoparticles
Source: Nat Commun. 2015 Dec 4;6:10101. doi: 10.1038/ncomms10101 (PMC4686773; doi:10.1038/ncomms10101)
Supplement: Supplementary Information — Supplementary Figures 1-11, Supplementary Tables 1-2, Supplementary Methods and Supplementary Reference [file ncomms10101-s1.pdf]

Supplementary Figures

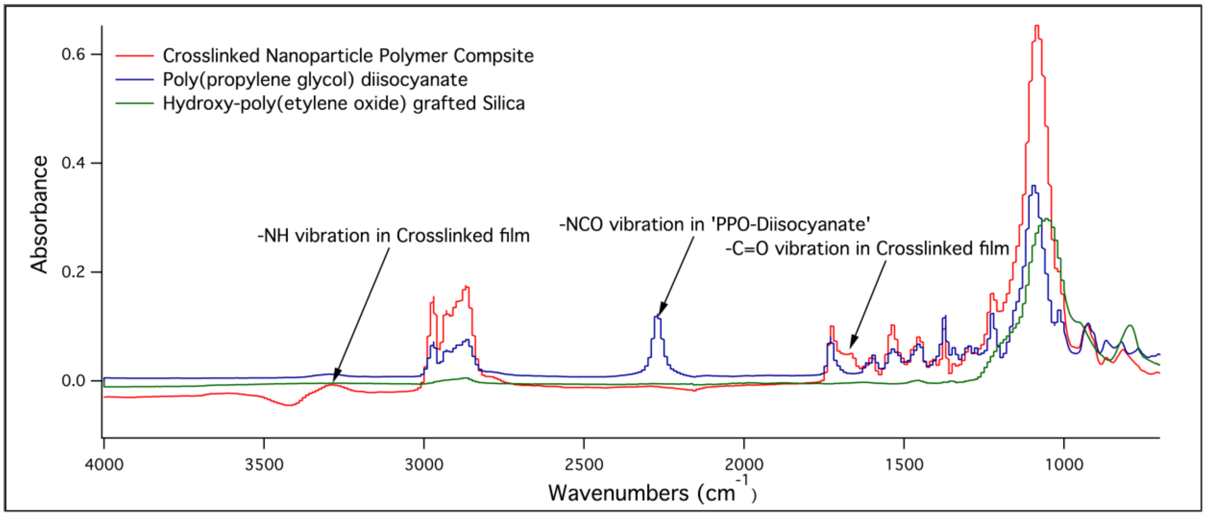

Supplementary Fig. 1: Fourier Transform- Infrared Spectroscopy (FTIR) Characterization of the Reaction Scheme: The reaction process is confirmed by the IR-peak transition involved in urethane reaction. The –NCO peak of the PPO Diisocyanate disappears, while characteristic peaks of –NH vibration and shift in the –C=O peak appear because of the urethane bond formation in the final product.

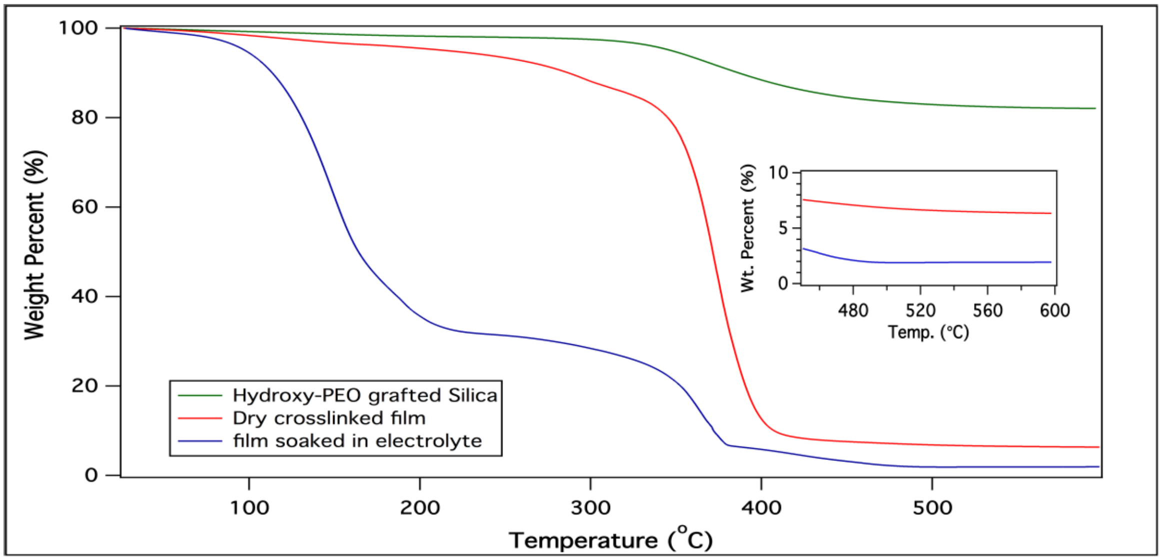

Supplementary Fig. 2: TGA Analysis: The initial silica content in the hairy nanoparticle (Si-PEO) was 83%, which reduces to 6% silica in the final product of crosslinked film. Thus, the non-conducting entity in the entire material is remarkably low, in-spite of having decent mechanical strength. Further soaking the CNPC in 1M electrolytes comprising of PC-LiTFSI, reduces the silica content close to 2%, as shown in the inset of the figure. Also, it is evident that the film is remarkably stable, such that there is degradation of the product up-to 120°C, where PC starts to degrade.

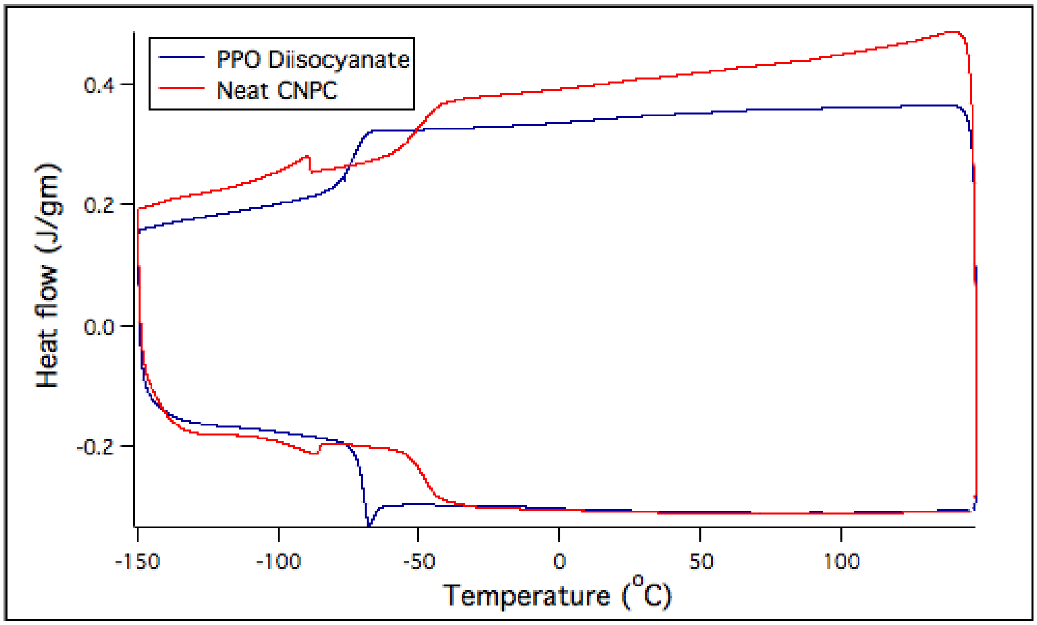

Supplementary Fig. 3: DSC Characterization of: The glass transition temperature of the PPO polymer increases from -63°C to -42°C due to the reduction of free volume as the chains are constricted due to the crosslinking. However, the crosslinked film is still in amorphous state at room temperature where it is used as electrolyte in battery systems. The amorphous nature of the polymer membrane enables higher ionic conductivity compared to other high MW PEO based electrolyte at room temperature.

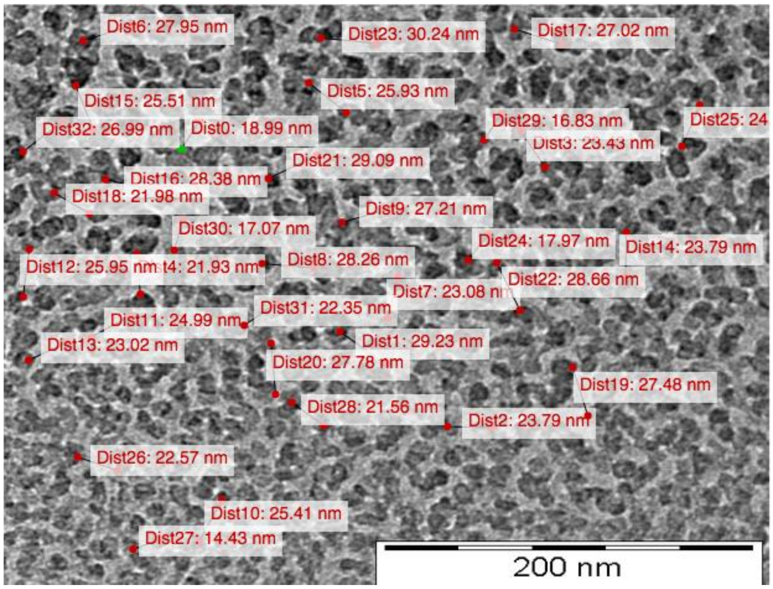

Supplementary Fig. 4: TEM Analysis: The inter-particle spacing in the crosslinked polymer is estimated by graphical analysis of TEM micrograph.

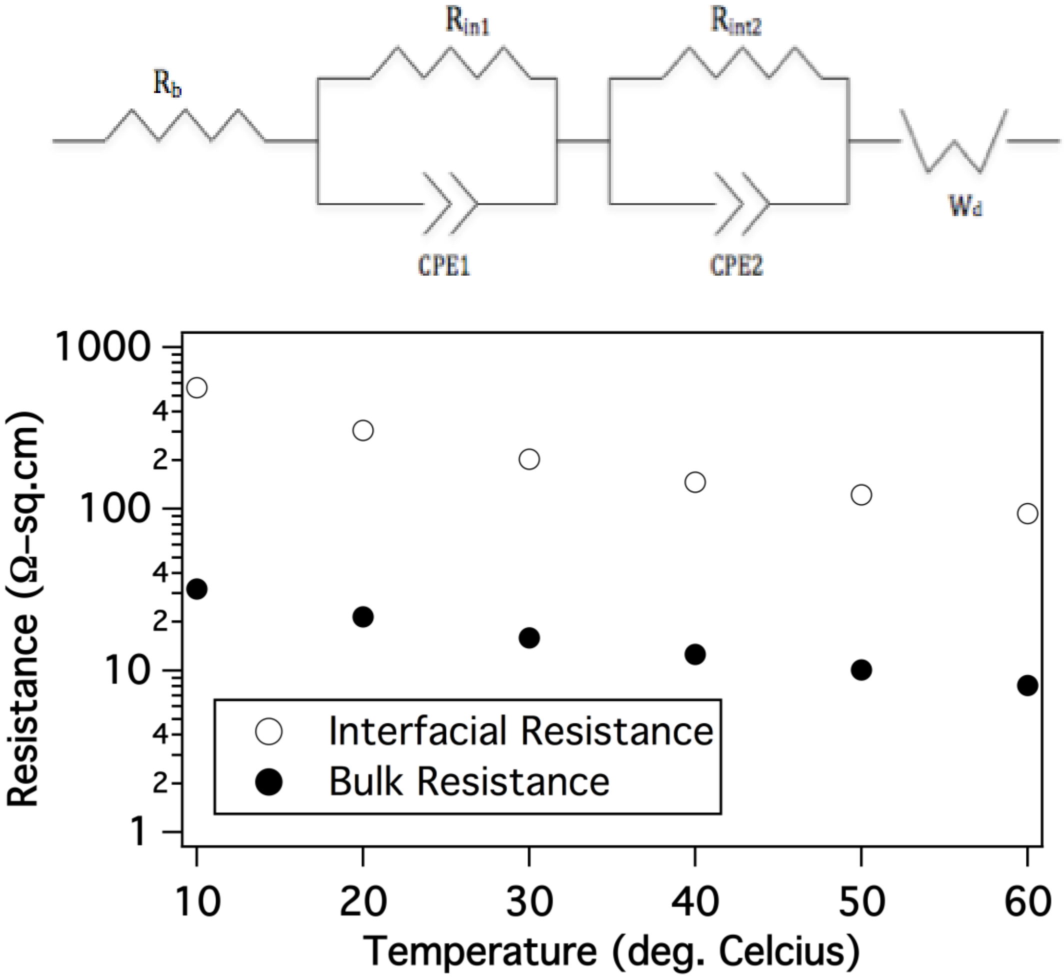

Supplementary Fig. 5: Equivalent Electric Circuit for the Impedance Spectroscopy results: The Nyquist plot obtained by the measurement of the Impedance at a wide range of frequency can be fitted by an equivalent circuit shown above. The bulk resistance and the interfacial resistance, thus obtained are plotted against temperature; and it is seen that the interfacial resistance is always higher than the bulk resistance, which indicates interface limited ion transfer.

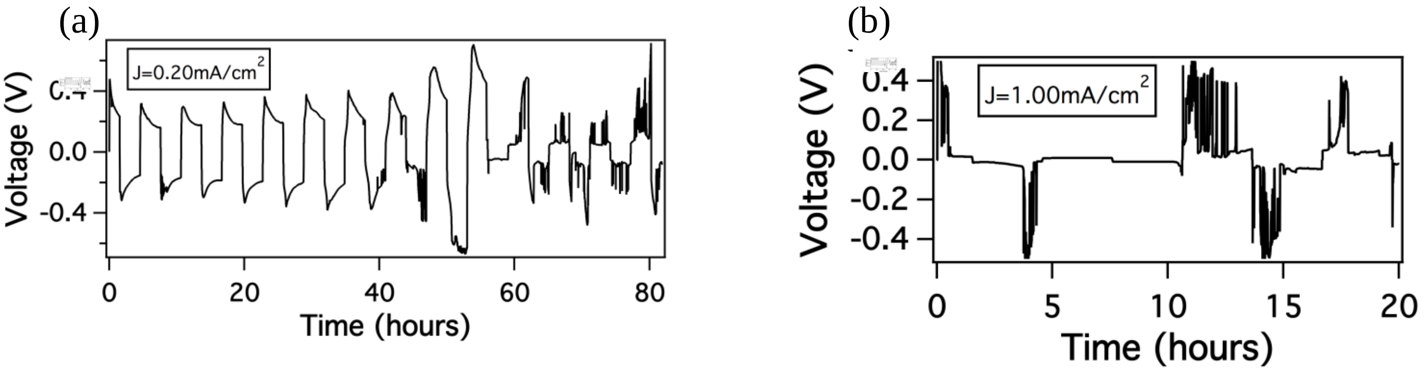

Supplementary Fig. 6: Strip-Plate measurement of a symmetric Lithium cell without CNPC separator: The voltage profile of a control cell (Li/PC+LiTFSI/Li) is plotted against time. **a,** it is seen that at current density of 0.20mA/cm2 about 55 hours of charging-discharging, the voltage profile gets distorted and the voltage range drops down which is a signature of dendrite induced short circuit. **b,** at current density of 1.00mA/cm2, the voltage profile is unstable even at the start of cycle.

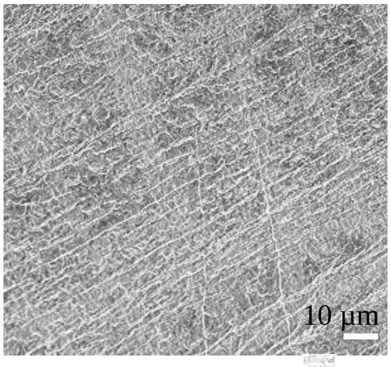

Supplementary Fig. 7: SEM image of Pristine Lithium: Surface of Lithium is presented in order to compare the changes in surface morphology after cycling using neat and crosslinked gel based electrolytes. (Scale bar is 10 microns)

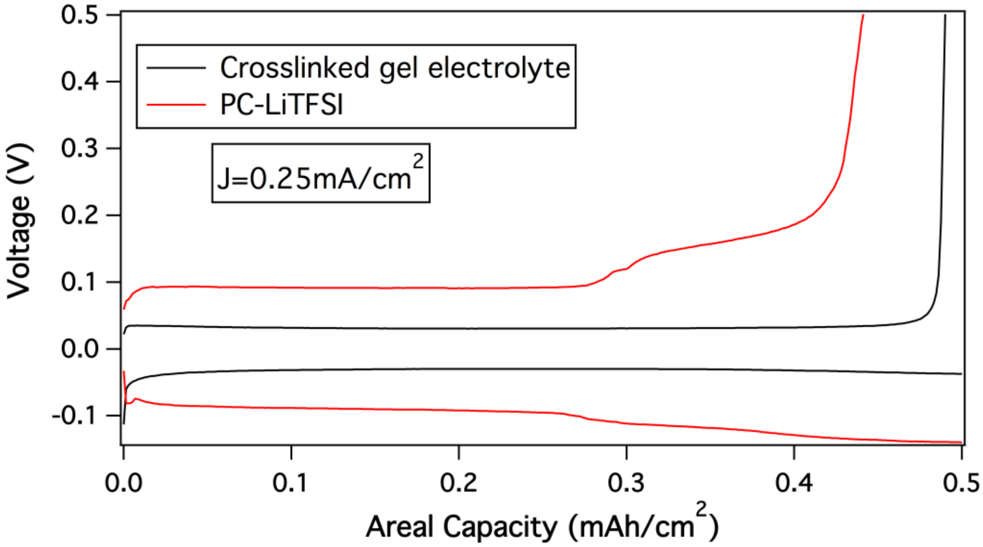

Supplementary Fig. 8: Coulombic Efficiency Test using Li| electrolyte| Stainless Steel configuration showing 40th cycle: Batteries with crosslinked gel electrolyte and pristine PC-LiTFSI were cycled at 0.25mA/cm^2. It is seen that at the 40th cycle, the neat electrolyte exhibits deposition at a much lower voltage and also low coulombic efficiency compared to the crosslinked gel electrolyte.

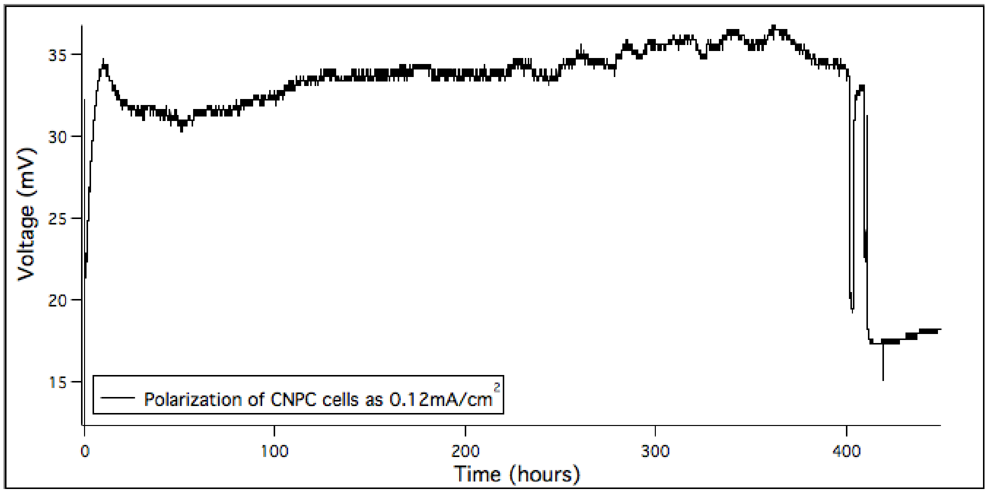

Supplementary Fig. 9: Polarization curve of symmetric Lithium cell with crosslinked gel electrolyte: A symmetric Lithium cell consisting of crosslinked gel electrolyte is charged constantly at a current density of 0.12mA/cm^2. It is seen that the cell successfully depositing Li ion onto the anode surface for about 400 hours before failing, indicated by the drop in voltage profile.

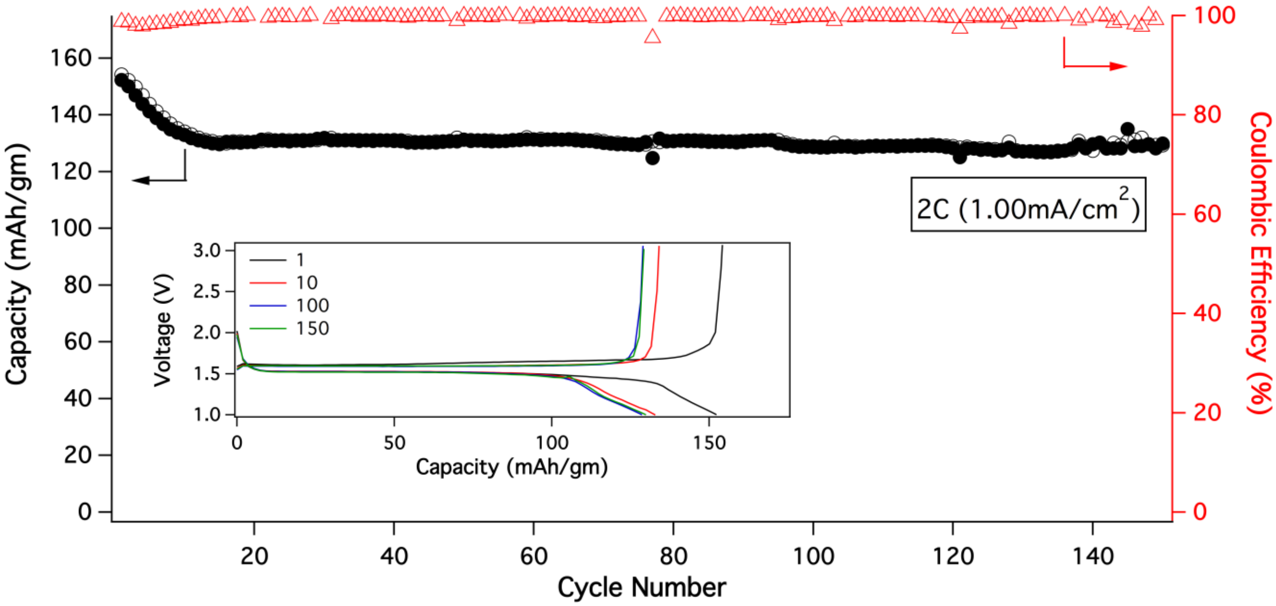

Supplementary Fig. 10: **Cycling Performance using LTO cathode:** It is seen that using the crosslinked gel electrolyte, a LTO based battery cycles well for over 150 cycles at a high current density of 1mA/cm^2

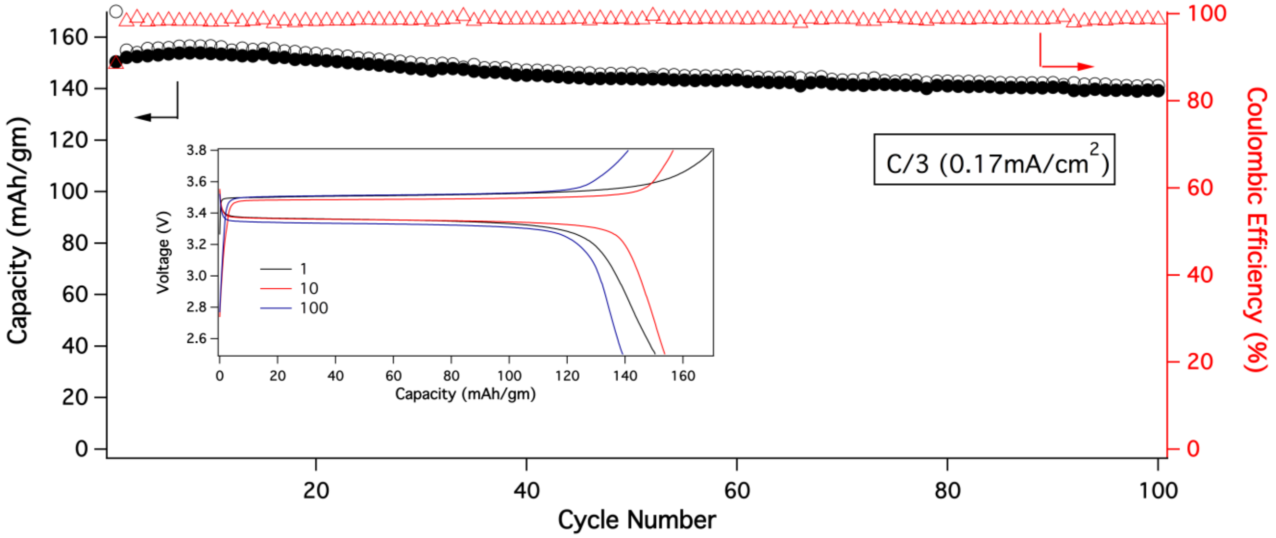

Supplementary Fig. 11: **Cycling Performance of $LiFePO_4$ battery using crosslinked gel electrolyte:** It is seen that at a C-rate of C/3, the battery cycles with minimum fade for at least 100 cycles.

Supplementary Tables

Type of product	Si-PEO	Si-PEO-PPO	Si-PEO-PPO-Electrolyte
**Component**	**Wt. %**	**Wt. %**	**Wt. %**
Silica	83	6	2
PEO	17	1.3	0.4
PPO	-	92.7	30.9
Electrolyte	-	-	66.7

Supplementary Table 1: Content of different components at successive synthesis stage: The weight percent of the Silica, PEO, PPO, electrolyte is given in table. It is seen that ultimately in the crosslinked gel electrolyte contains as low as 2% silica, still having a relatively high mechanical modulus.

Parameters	A (S/cm)	B (K)	T_O (K)
LiPF6-EC/DEC	0.045	56.9	237
LiTFSI-PC	0.044	61	239
Crosslinked film-LiPF6-EC-DEC	0.239	870.5	84.3
Crosslinked film-LiTFSI-PC	0.237	891.5	85

Supplementary Table 2: VFT parameters of the different electrolyte configuration: $\sigma = A\exp(-B/(T-T_O))$, where A is the pre-exponential factor corresponding to conductivity at infinite temperature, B is the Activation Energy and T_O is the reference temperature.

Supplementary Methods:

The size of clusters was obtained by fitting the SAXS data with Beaucage unified equation [1] as shown below.

$$I(q) = A\exp\left(-\frac{q^2R_p^2}{3}\right) + Berf\left(\frac{\left(\frac{qR_p}{\sqrt{6}}\right)^3}{q}\right)^{p_1} + \sum_i C_i \exp\left(-\frac{q^2R_p^2}{3}\right)erf\left(\frac{\left(\frac{qR_{c,i}}{\sqrt{6}}\right)^3}{q}\right)^{p_{2,i}}$$

First two terms (Guinier and power law) contribute to the scattering for spheres in a dilute suspension with radius $a = \sqrt{\frac{5}{3}}R_p$ ~ 4-5 nm and power law exponent p_1 (~4). A and B are the Guinier and Porod scaling factors. The last term contributes to the scattering from the fractal objects in the low *q* regime with $R_{fractal} = \sqrt{\frac{5}{3}}R_c$ ~ 51-72 nm with a power exponent p_2 ~2, indicating the fractals to be mass fractals. Since the low q regime has only the power law scattering no Guinier term has been included suggesting that the $R_{fractal}$ obtained from the fitting will be the lower bound as exact dimension cannot be determined. C is the pre-factor factor for the power law scattering in the low *q*. Absence of any additional structure contribution in intermediate to high *q* suggests that the particles are reasonably far apart.

Supplementary Reference

1. Beaucage, G., Small-angle scattering from polymeric mass fractals of arbitrary massfractal dimension. *Journal of Applied Crystallography* **29** (2), 134-146 (1996)
